# Diseases of the Heart and Circulation

**Published:** 1902-01-11

**Authors:** 


### Diseases of the Heart and Circulation.

**Nauheim Mineral Waters.**—Robertson[1] has published an important article on the use of the mineral waters of Nauheim, based on an extensive personal experience both on himself and on patients. The chief constituents of the waters are common salt, chloride of calcium, and carbonic acid gas. No. 12 spring is richest in salts, containing 3 per cent. of salt and $\frac{1}{4}$ per cent. of chloride of calcium, while No. 7 contains $2\frac{1}{5}$ per cent. of the former and $\frac{1}{6}$ per cent. of the latter salt; the new spring No. 14 coming between the two. No. 7 spring is richest in gas, and is of a lower temperature.

If a patient with evidence of cardiac dilatation and failure step into such a bath, he will at the end of a minute say he is very uncomfortable, his pulse will be harder and slower, and his cyanosis perhaps a little greater. But very shortly, in, say, another minute, his colour improves, breathing is deeper and easier, his pulse fuller and firmer, and while much slower than when the patient stepped in, yet not so slow as during the first minute. The patient gets more and more easy, and when he leaves the bath, in, say, 15 minutes, is a very different man from the one who entered it. It may be noticed that his skin is of a uniform pink colour where it was covered by the water. This vaso-motor dilatation in the skin must have a very direct effect on the heart itself, by lessening the peripheral resistance and by unloading the internal organs. Other phenomena are also noticeable; (1) slowing of the heart beat; (2) strengthening of the heart beat; (3) diminution in the area of cardiac dulness; and (4) changes in the blood pressure, in the direction of heightening or lowering according to other concomitant circumstances.

The effect then is not simply one of an increased peripheral resistance raising the blood pressure and so slowing the heart. The blood pressure may either be lowered or heightened. There are two effects—(1) the chemical action on the skin producing vasodilatation; and (2) the reflex action on the heart. By the first the work of the heart is lessened, and by the other its ability to do its work is increased. This contraction of the heart has been shown, not merely by the diminution in the cardiac dulness, but also by the Roentgen rays. The kind of cases likely to benefit by the use of the waters may be arranged into the following broad classes: (1) It is to be remembered that the Nauheim baths first achieved a reputation for the treatment of gout, rheumatism and scrofula. Its value in these diseases probably depends mainly on the marked changes the baths produce in the distribution of blood and the quickening of tissue metamorphosis thereby induced. This action of the baths is a little apt to be lost sight of. (2) The second type of case is that of muscular weakness of the heart, due to physical overstrain, prolonged overwork, or the result of influenza or similar toxic influence, *e.g.*, rheumatism. This is the class of case which exhibits the benefits of the bath treatment in the highest degree. (3) The third type is the case of valvular disease in which compensation has failed or has never been properly established. In such cases owing to the strengthening of the cardiac muscle the abnormal sounds may be actually intensified. (4) The fourth type of disease is that of the cardiac neuroses, *e.g.*, vaso-motor angina. In these the results are often brilliant.

The Schott methods of resisted movements are often added to the baths with great advantage. They are movements of flexion and extension, abduction and adduction, of groups of muscles, performed against a gentle resistance made by an assistant. The pulse is the guide both for the degree of resistance and the number of movements given at each séance. Should the pulse begin to rise the exercise is stopped. The effect of these movements is a dilatation of the muscular arterioles, and a reflex effect on the heart, the result depending on the degree and severity of the exercise. If it be gentle there is a lowering of blood pressure and a slowing of the heart-beat. The exercises thus develop and invigorate the muscle of the heart and form a therapeutic method of great value. Robertson ends by giving the quantities of ingredients necessary to make the several kinds of baths for use in this country.

Leusser [2] has been employing, at Kissengen, methods similar to those in use at Nauheim. The baths are of water containing carbon dioxide and brine; they are given in gradually increasing strength, and their temperature is gradually diminished (from 95° to 88° F.), according to the reaction of the patient. Leusser ascribes the action of the baths (1) to the intense stimulation of the peripheral nerve endings by the $CO_2$ bubbles spread over the whole body, and (2) to the dilatation of the vessels of the skin (after temporary constriction) by the chemical and thermal stimulation of the baths, so that the heart works more freely. Certain improvement, and even cure may be expected in neuroses of the heart (palpitation, neurasthenia cordis), overworked heart with or without slight dilatation, heart weakness due to irregular living, to nicotine, alcohol, coffee, in the heart oppressed by fatty overgrowth, in dilatation due to anæmia and chlorosis, in endocarditis after the acute stage has been passed.

Ballistini and Rovere [3] have made experiments, with artificially prepared baths, to test the question, which as yet is unsettled, whether there is an increase or a diminution in the blood pressure as a result of the baths. They found in most cases a considerable increase of blood pressure, and in cases in which a diminution occurred, this was only temporary. The increase was always proportionate to the concentration of the bath, and especially to the amount of $CO_2$. In cases of aortic incompetence with attacks of angina pectoris, the baths did harm in spite of a fairly high temperature. In such cases and in profound arterio-sclerosis the baths are contra-indicated. The pulse was always slowed after each bath by 12–20 beats, and the slowing lasted several hours, and to a certain extent remained as a permanent result of the treatment. There was no constant relation between pulse and blood pressue. Arhythmia, when present, tended to become less. In most cases subjective symptoms were relieved, especially dyspnœa and palpitation. In severe cases with marked failure of compensation, the treatment was quite inadequate, but in heart weakness following rheumatism or other infection, it may be useful. The best results are obtained when no organic disease of the heart is present, and in failure of the valves, especially in failure of the mitral valves. Heineman [4] and Wakefield [5] have also recently written papers on the Nauheim treatment, covering much the same ground as those just abstracted,

[1] Edin. Med. Jour., 1901., Vol. IX., p. 513, and Vol. X., p. 20. [2] Münch. Med. Wochenschrift, 1901, pp. 744 and 792. [3] Zeitsch. f. Diätet u. Physikal. Therap., 1901, p. 552. [4] Med. Rec., June 22, 1901. [5] Med. Rec., Sept. 14, 1901.

